# Immune microenvironment spatial landscapes of tertiary lymphoid structures in gastric cancer

**DOI:** 10.1186/s12916-025-03889-3

**Published:** 2025-02-04

**Authors:** Yi Xie, Haoxin Peng, Yajie Hu, Keren Jia, Jiajia Yuan, Dan Liu, Yanyan Li, Xujiao Feng, Jian Li, Xiaotian Zhang, Yu Sun, Lin Shen, Yang Chen

**Affiliations:** 1 Department of Gastrointestinal Oncology, Key Laboratory of Carcinogenesis and Translational Research (Ministry of Education, Beijing), Peking University Cancer Hospital and Institute, Beijing, 100142 China; 2 Department of Pathology, Key Laboratory of Carcinogenesis and Translational Research (Ministry of Education), Peking University Cancer Hospital and Institute, Beijing, 100142 China; 3 Department of Gastrointestinal Cancer, Beijing GoBroad Hospital, Beijing, 102200 China

**Keywords:** Gastric cancer, Tertiary lymphoid structures, Tumour microenvironment, Immunotherapy, Prognostic model, Single-cell RNA sequencing, Spatial transcriptomics

## Abstract

**Background:**

Tertiary lymphoid structures (TLS) correlate with tumour prognosis and immunotherapy responses in gastric cancer (GC) studies. However, understanding the complex and diverse immune microenvironment within TLS requires comprehensive analysis.

**Methods:**

We examined the prognostic impact of TLS within the tumour core (TC) of 59 GC patients undergoing immunotherapy. Multispectral fluorescence imaging was employed to evaluate variations in immune cell infiltration across different TLS sites among 110 GC patients, by quantifying immune cell density and spatial characteristics. We also generated a single-cell transcriptomic atlas of TLS-positive (*n* = 4) and TLS-negative (*n* = 8) microenvironments and performed spatial transcriptomics (ST) analysis on two samples.

**Results:**

TLS presence in the TC significantly correlated with improved immune-related overall survival (*P* = 0.049). CD8^+^LAG-3^−^PD-1^+^TIM-3^−^, CD4^+^PD-L1^+^, and CD4^+^FoxP3^−^ T cell densities were significantly higher in the TLS within TC compared to tumour and stromal regions. Immune cells within TLS exhibited closer intercellular proximity than those outside TLS. Five key density and spatial characteristics of immune cells within TLS in the TC were selected to develop the Density and Spatial Score risk model. Single-cell RNA sequencing revealed strong intercellular interactions in the presence of TLS within the microenvironment. However, TLS-absent environment facilitated tumour cell interactions with immune cells through MIF- and galectin-dependent pathways, recruiting immunosuppressive cells. ST analysis confirmed that T and B cells co-localise within TLS, enhancing immune response activation compared to cancer nests and exerting a strong anti-tumour effect.

**Conclusions:**

TLS presence facilitates frequent cell-to-cell communication, forming an active immune microenvironment, highlighting the prognostic value of TLS.

**Supplementary Information:**

The online version contains supplementary material available at 10.1186/s12916-025-03889-3.

## Background

Gastric cancer (GC), one of the most prevalent cancers and the second leading cause of cancer-related deaths worldwide, is a major global health concern [1]. GC cases are frequently diagnosed at advanced stages, characterised by an unfavourable prognosis and considerable resistance to conventional therapeutic modalities [2]. Immunotherapy is a promising treatment for GC, emphasising the need to understand mechanisms that enable robust antitumour immune responses.

Tertiary lymphoid structures (TLS) are ectopic lymphoid formations that develop in non-lymphoid organs in response to chronic inflammatory stimuli, such as infections and tumours, playing a pivotal role in the antitumour immune response within the tumour microenvironment (TME). The presence of intratumoural TLS correlates with a favourable prognosis in several cancers such as breast cancer [3], lung cancer [4], sarcoma [5], and oesophageal squamous cell carcinoma [6]. Typically identified through haematoxylin and eosin (H&E) staining of tissue slides, TLS are found in the tumour core (TC), at the invasion margin (IM) or surrounding peri-cancerous normal (N) areas. In hepatocellular carcinoma, the presence of peritumoural TLS is linked to an increased risk of cancer recurrence and adverse outcomes compared with those of intratumoural TLS [7]. Studies assessing the predictive power of TLS for the effectiveness of immune checkpoint inhibitors in GC have not adequately considered the specific location of TLS. The influence of TLS location heterogeneity on the GC immune microenvironment requires urgent investigation.

TLS consist of organised lymphoid aggregates containing a variety of immune cells [8]. Precise identification of specific tumour-immune cell subpopulations associated with TLS requires the use of multiple markers. Multiplex immunohistochemistry (m-IHC) enables the detection of the types and spatial arrangements of tumour-infiltrating immune cells (TIICs) within the TLS at single-cell resolution, providing a detailed view of the TLS internal structural characteristics. Within TLS, immune cells interact with each other and non-immune cells in the surrounding tissue, fostering a localised immune response against cancer. Interaction between B cells and CXCL13^+^CD103^+^CD8^+^ tissue-resident memory T cells in TLSs promotes an antitumour immune response in GC [9]. However, the functions of other cell types in the TLS are yet to be explored. By integrating single-cell RNA sequencing (scRNA-seq) and spatial transcriptomics (ST) analyses, the composition and relative abundance of different cell types in the TME were established. This approach has facilitated a deeper understanding of the roles of various immune cells in TLS, their interactions with adjacent tumour cells, and the dynamics of intercellular communication and signalling within the TLS context.

In this study, we first examined the prognostic significance of the TLS within the TC through a morphological examination of 59 patients who received immunotherapy. We used m-IHC to identify TLS in GC clinical specimens and delineate the immune cell subtypes within them. We characterised the density and spatial distribution of TIICs by examining variations in TLS locations and their prognostic implications. These observations were utilised to construct a prognostic model. Then, scRNA-seq and ST analyses of TLS in GC were employed to delve into the role of TLS in the immune microenvironment of GC and its effects on tumour cell functionality.

## Methods

### Patients and samples

Patients were recruited from the Department of Gastrointestinal Oncology between 1 January 2016 and 1 June 2020. The inclusion criteria specified that individuals must be pathologically diagnosed with primary gastric adenocarcinoma and have adequate formalin-fixed and paraffin-embedded (FFPE) tissue blocks available for analysis. Patients with autoimmune diseases, HIV infection, or syphilis were excluded from the current study. This study was approved by the Ethics Committee of the Peking University Cancer Hospital (2024KT56). Written consent was obtained from all patients or their legal guardians prior to sample collection.

**Fig. 1:**
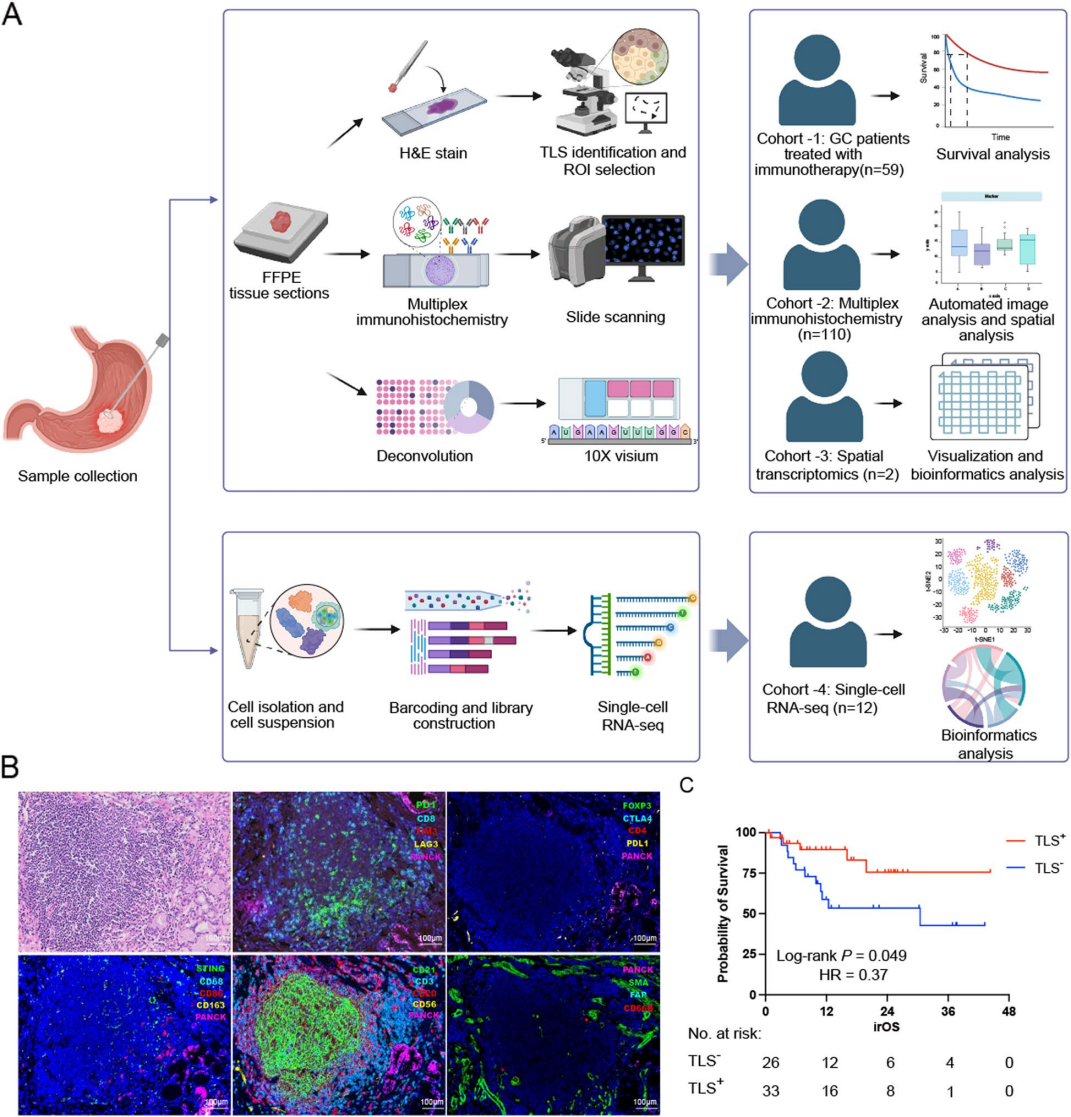
Schematic diagram of the workflow of the study. **A** Schematic representation of the experimental design and analytical methods used in this study. **B** Representative composite images of TLS from the multiplex immunohistochemistry panels used. Scale bar: 100 μm. **C** Kaplan–Meier survival curves for irOS of patients with and without TLS in the tumour core. *p*-values are two sided. TLS, tertiary lymphoid structure; irOS, immune-related overall survival

Four distinct cohorts were identified (Fig. 1A). For the survival analysis, Cohort -1 consisted of 59 patients who underwent anti-PD-1/PD-L1 therapy at an advanced stage. The treatment regimen was immunotherapy combined with chemotherapy. The detailed clinicopathological characteristics of the patients are summarised in Table 1. Among them, 16 patients were initially diagnosed with stage I-III gastric cancer and subsequently developed distant metastases. The detailed distribution of metastases in stage I–III patients are summarised in Additional file 2: Table S1. Cohort -2, comprising 110 GC specimens, was subjected to m-IHC analysis to elucidate the immune landscape. ST analysis was performed in two GC patients (Cohort -3). scRNA-seq was conducted on 12 GC specimens obtained from 12 GC patients (Cohort -4). In detail, four samples exhibiting TLS were categorised as TLS^+^ group, while the remaining eight samples lacking TLS were designated as the TLS^−^ group. Comprehensive information on the clinical characteristics of Cohort -2, -3, and -4 is provided in Additional file 2: Table S 2–4.

**Table 1:** The baseline characteristics of 59 gastric adenocarcinoma patients

Characteristic	Total (*N* = 59)	TLS − (*N* = 26)	TLS + (*N* = 33)	*P* value
Age at diagnosis				0.706
< 65	37 (62.7)	17 (65.4)	20 (60.6)	
≥ 65	22 (37.3)	9 (34.6)	13 (39.4)	
Gender				0.917
Male	45 (70.7)	20 (76.9)	25 (75.8)	
Female	14 (29.3)	6 (23.1)	8 (24.2)	
Stage at diagnosis				0.863
I	2 (3.4)	1 (3.8)	1 (3.0)	
II	3 (5.1)	2 (7.7)	1 (3.0)	
III	11 (18.6)	5 (19.2)	6 (18.2)	
IV	43 (72.9)	18 (69.2)	25 (75.8)	
Tumour differentiation				0.006
High	7 (11.9)	6 (23.1)	1 (3.0)	
Moderate	22 (37.3)	13 (50.0)	9 (27.3)	
Poor	27 (45.8)	6 (23.1)	21 (63.6)	
Unknown	3 (5.1)	1 (3.8)	2 (6.1)	
Lauren classification				0.226
Intestinal type	24 (40.7)	13 (50.0)	11 (33.3)	
Mixed type	15 (25.4)	6 (23.1)	9 (27.3)	
Diffused type	18 (30.5)	5 (19.2)	13 (39.4)	
Unknown	2 (3.4)	2 (7.7)	0 (0.0)	
Location				0.211
GEJ	22 (62.7)	14 (53.8)	23 (69.7)	
Non-GEJ	37 (37.3)	12 (46.2)	10 (30.3)	
HER2 expression				0.265
Positive	12 (20.3)	7 (26.9)	5 (15.2)	
Negative	47 (79.7)	19 (73.1)	28 (84.8)	
EBER expression				0.689
Positive	4 (6.8)	2 (7.7)	2 (6.1)	
Negative	52 (88.1)	22 (84.6)	30 (90.9)	
Unknown	3 (5.1)	2 (7.7)	1 (3.0)	
Microsatellite status				0.120
Microsatellite instability (MSI)	7 (11.9)	5 (19.2)	2 (6.1)	
Microsatellite-stable (MSS)	52 (88.1)	21 (80.8)	31 (93.9)	
PD-L1 CPS				0.646
CPS < 1	24 (40.7)	10 (38.5)	14 (42.4)	
1 ≤ CPS < 5	10 (16.9)	6 (23.1)	4 (12.1)	
5 ≤ CPS < 10	4 (6.8)	1 (3.8)	3 (9.1)	
CPS > 10	18 (30.5)	7 (26.9)	11 (33.3)	
Unknown	3 (5.1)	2 (7.7)	1 (3.0)	
Line of therapy				0. 068
1	35 (59.3)	12 (46.2)	23 (69.7)	
≥ 2	24 (40.7)	14 (53.8)	10 (30.3)	

*Abbreviations*: *GEJ* gastro-oesophageal junction, *EGFR* epidermal growth factor receptor, *HER2* human epidermal growth factor receptor 2, *CPS* combined positive score, *PD-1* programmed cell death protein 1, *PD-L1* programmed death-ligand 1

Regarding tissue sampling, all samples were collected before systemic therapy. None of the patients had received neoadjuvant therapy. Sampling was consistently performed from the primary GC lesion.

### TLS identification and regions of interest (ROIs) selection

Two pathologists specialising in professional pathology independently reviewed the tumour tissue sections and were blinded to the clinical data. TLS evaluation and ROIs selection were performed retrospectively using H&E staining. All H&E slides were evaluated for TLS using digital WSIs, which were acquired at Peking University Cancer Hospital using 3DHISTECH slide converter 2.3. All sections of each patient’s tumour were examined to comprehensively assess TLS status, requiring that TLS diameters exceed 200 microns. The ROI selection was conducted across the full field of view and categorised into three distinct regions as follows: TC, IM (areas 1 mm inward and outwards from the tumour boundary), and normal tissue adjacent to the tumour (N).

### Multiplex immunohistochemistry

We performed multiplex IHC on five panels of tumour tissues to determine the expression level and spatial distribution of CD8, PD-1, TIM-3, LAG-3, CD4, FoxP3, CTLA-4, PD-L1, CD68, CD163, CD86, STING, CD20, CD21, CD3, FAP, and CD66b. For Cohort -2, the samples were collected within 30 min of tumour removal, fixed in 10% formalin for 24–48 h, dehydrated, and embedded in paraffin. Six consecutive sections, each with a thickness of 4 μm, were cut from paraffin blocks. One section was set aside for H&E staining to evaluate the morphological features. Five 4 μm FFPE tumour slides were melted at 60 °C for 12 h to dehydrate, then de-paraffinised in xylene and rehydrated using graded alcohol solutions. Antigen retrieval was meticulously performed using a microwave with either ethylenediaminetetraacetic acid buffer (pH 9.0) or citrate buffer (pH 6.0), tailored for FoxP3 staining. Then, sections were blocked with a commercial blocking buffer (Dako, Santa Clara, CA, USA; cat. X0909) for 10 min to prevent non-specific binding.

Additional file 2: Table S5 lists the antibodies and their manufacturers used for staining. Following a defined sequence of staining and the pre-optimised antibody concentration, the slide specimens were procedural incubated with the primary antibody, followed by horseradish peroxidase-conjugated secondary antibody application, after which tyramine signal amplification (TSA) was conducted. Each TSA application was successful for antibody stripping and antigen retrieval. Subsequently, the cellular nuclei were distinctively stained utilising 4′,6-diamidino-2-phenylindole (DAPI, Sigma-Aldrich, St. Louis MO, USA; cat. D9542).

### Multispectral imaging

Images were captured through whole-slide scanning utilising an Olympus VS200 MTL (Olympus, Germany) and an Olympus UPLXAPO × 20 objective lens. The images were viewed using OLYMPUS OlyVIA 3.3 software. Initially, pathologists marked the ROIs using H&E-stained images. Data processing was supervised by a pathologist to ensure quality.

**Fig. 2:**
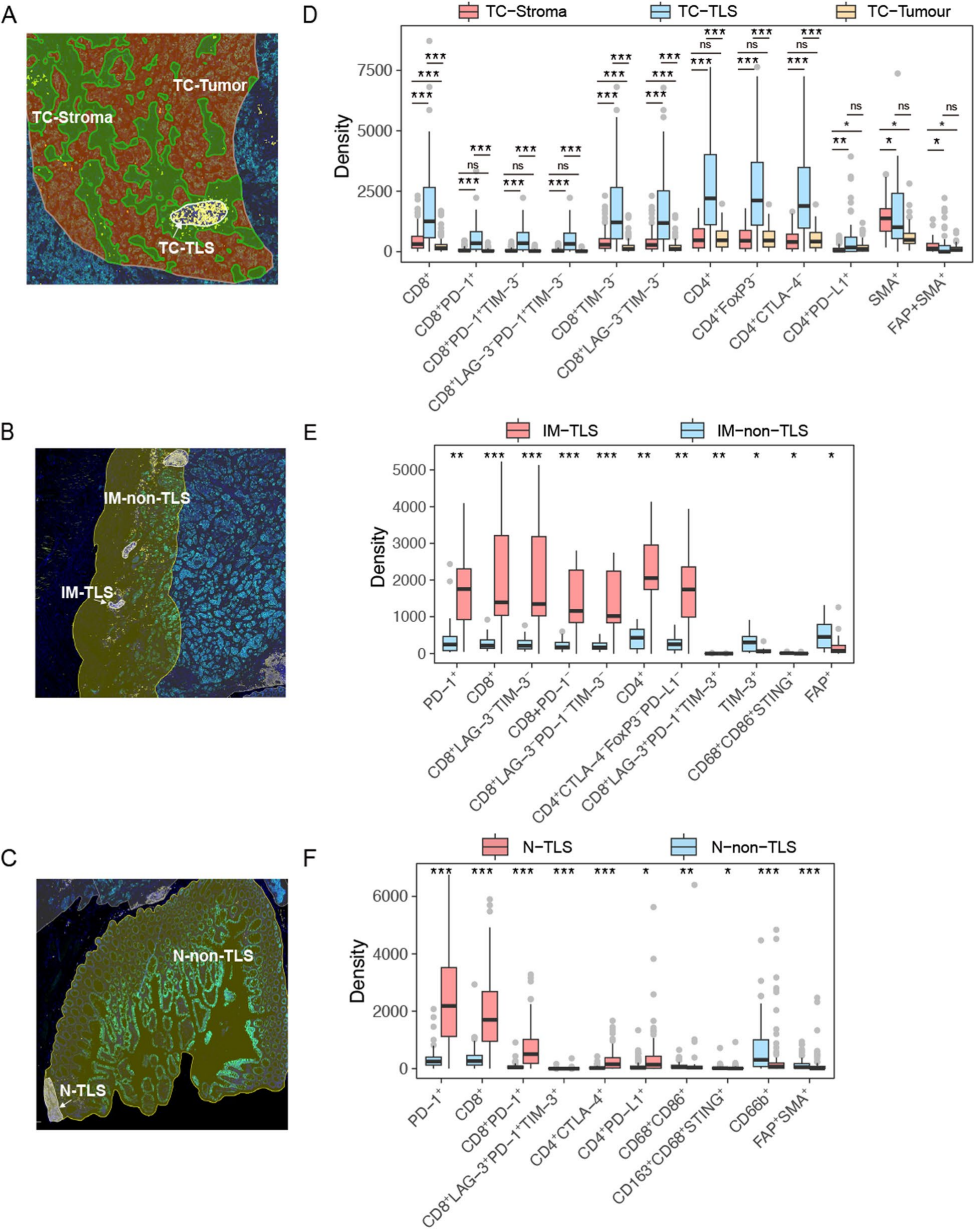
Spatial heterogeneity of TLS influences immune cell density in the tumour microenvironment. **A**–**C** Representative images of tumour core (**A**), invasion margin (**B**), and normal region (**C**). TLS, tertiary lymphoid structure; TC-TLS, TLS in the tumour core; TC-Stroma, stroma regions in the tumour core; IM-TLS, TLS in the invasion margin; IM-non-TLS, invasion margin excluding TLS; N-TLS, TLS in the normal tissue; N-non-TLS, normal tissue excluding TLS. **D**–**F** Density of tumour-infiltrating immune cells (TIICs) of tumour core (**D**), invasion margin (**E**), and normal tissue (**F**). Box and whiskers represent mean ± 10–90 percentile. Three groups were compared: Kruskal–Wallis test with Dunn’s multiple comparison test. Two groups were compared: Wilcox test. Each point represents one patient. *p < 0.05, **p < 0.01, ***p < 0.001 and not significant (ns)

The presence of TLS was further confirmed through a multiplex immunofluorescence assay using the markers CD4, CD8, CD3, CD20, and CD21 based on scales from prior research [10, 11]. At the cellular level, the TLS consists of an external T-cell zone (marked by CD3, CD4, or CD8) and a central B-cell zone comprising follicular dendritic cells expressing CD21 and numerous B cells marked by CD20. The requirement for TLS identification included a diameter greater than 200 microns, ensuring a rigorous evaluation of TLS within the studied tissues. Evaluations of the TLS within the TC, IM, and N regions were conducted separately (Additional file 1: Fig. S1A). TLS within the TC were termed TC-TLS, those within adjacent normal tissue were labelled N-TLS, and TLS at the IM were identified as IM-TLS. Regions outside the TLS within the IM and adjacent normal tissue areas were defined as the IM-non-TLS and N-non-TLS, respectively (Fig. 2A–C).

### Imaging data analysis

Whole-slide images were analysed using QuPath, v0.4.3. An algorithm was constructed through the steps of selection, training, and confirmation in the QuPath, v0.4.3, software to segment the tissue into three organisational categories: TC-Tumour, TC-Stroma, and TC-TLS. The area and number of each tissue type were recorded. Cells were precisely detected and counted using the QuPath software with a reasonable threshold set to identify positive cells. The R script (Version 4.2.1) data analysis software was then used to summarise the total and positive cell count and to derive the density of positive cells (where $Density=\frac{\sum \text{C}\_\text{P}}{\text{Area}}$, C_P represents the count of target phenotype cells, and Area is the area of the specific ROI being analysed).

### scRNA-seq sample collection and preparation

The fresh tumour tissues were preserved in GEXSCOPE® Tissue Preservation Solution (Singleron) and promptly transported to the Singleron laboratory on ice. Following transportation, the specimens were washed three times with Hanks Balanced Salt Solution before being minced into 1–2-mm pieces. Subsequently, the tissue fragments were enzymatically digested using 2 mL of GEXSCOPE® Tissue Dissociation Solution (Singleron) at 37℃ for 15 min within a 15-mL centrifuge tube with sustained agitation. Following digestion, the samples were filtered through 40-micron sterile strainers and centrifuged at 1000 rpm for 5 min. The pellet was resuspended in 1 mL of phosphate-buffered saline (PBS; HyClone), treated with 2 mL of GEXSCOPE® red blood cell lysis buffer (Singleron) at 25 °C for 10 min, centrifuged at 500* g* for 5 min, and resuspended in PBS. Finally, samples were stained with trypan blue (Sigma-Aldrich) for microscopic evaluation.

### Sample preparation process for scRNA-seq

Single-cell suspensions were prepared at 1 × 10^5^ cells/mL in PBS (HyClone) and loaded into microfluidic devices. ScRNA-seq libraries were then constructed following the Singleron GEXSCOPE® protocol, utilising the GEXSCOPE® Single-Cell RNA Library Kit (Singleron Biotechnologies) [12]. The libraries were diluted to 4 nM, pooled, and sequenced on an Illumina HiSeq X platform to generate 150 bp paired-end reads.

### Primary analysis of raw read data

Raw reads were processed using a standard internal pipeline to derive the gene expression profiles. Initial processing involved filtering read 1 to remove poly T tails and extracting cell barcodes and unique molecular identifiers (UMIs). Following the trimming of adapters and poly (A) tails using FASTP V1, read two was aligned to the GRCh38. This alignment was conducted using Ensemble version 92 gene annotation (fastp 2.5.3a and featureCounts 1.6.2) [13]. Reads sharing identical cell barcodes, UMIs, and gene identifiers were consolidated to compute the UMI count per gene per cell using the “count” command. The resulting UMI count tables were then analysed using the Seurat package (version 4.3.0) in R software (version 4.2.1) for each individual sample [14].

### Quality control, clustering, and cell type annotation

We filtered out the low-quality cells with expressed genes > 5000 or < 200 genes, > 10% mitochondrial reads, or < 500 unique UMIs. After quality control, 52,382 high-quality single cells were used for downstream analysis. Expression data were normalised using the NormalizeData function, which facilitates the correction of technical variability without compromising biological information. We used FindVariableFeatures to calculate the top 2000 genes with the greatest variability for principal component analysis (PCA) analysis. Data were visualised on a two-dimensional map using Uniform Manifold Approximation and Projection (UMAP). A nonlinear dimensional reduction was performed to cluster cells sharing common features. Cluster annotations were performed using the Seurat function FindAllMarkers, in conjunction with a compendium of classical and widely accepted marker genes [15].

### Cell–cell communication analysis

The putative intercellular communication networks were assessed using the CellChat algorithm [16]. The analysis commenced with the loading of normalised counts into CellChat, adhering to the standard workflow, to identify crucial ligand and receptor pairs that facilitate cluster-specific communication. Subsequent preprocessing involved the identifyOverExpressedGenes, identifyOverExpressedInteraction, and ProjectData functions, all of which were set to default parameters. The dominant functional roles of specific cell types within the TME were ascertained by ranking ligand-receptor interaction networks based on information flow, employing the RankNet function.

### Differential expression and pathway analysis

Differentially expressed genes (DEGs) across distinct groups were delineated using the FindMarkers function [17]. Subsequently, gene set variation analysis (GSVA) function enrichment analysis was employed to elucidate functional and pathway divergences across various cell lineages [18]. The DEGs and pathways for each cell type were analysed using the limma package, adhering to the statistical threshold of a Bonferroni-corrected *p*-adjusted value (padj) of less than 0.05.

### Single-cell copy-number variation (CNV) evaluation

To identify malignant cells within solid tumours, the InferCNV tool (https://github.com/broadinstitute/inferCNV) was used to evaluate CNVs in somatic chromosomes [19]. The expression intensities of genes across genomic loci in epithelial cells (ECs) were compared to those in a set of reference T cells. Any genes exhibiting a mean count of 0.1 or below were systematically excluded. Within this framework, some cells were identified as malignant, whereas the remainder were classified as normal.

### ST sequencing and ST data processing

Prior to RNA extraction, formalin-fixed paraffin-embedded GC tissues were embedded in paraffin. Each tumour section, sliced to a thickness of 10 μm, was mounted onto ST microarrays for analysis. Subsequently, the tissues were dehydrated in isopropanol for 1 min and stained with haematoxylin and eosin. Bright-field photographs were captured at a × 20 magnification. Two experienced pathologists (H.Y.J. and S.Y.) then conducted a thorough examination of the H&E-stained slides, confirming the pathology and manually annotating distinct tissue regions. Library construction and sequencing were subsequently performed on the tissue sections using Visium Spatial Gene Expression Reagent Kits (10 × Genomics, Pleasanton, CA, USA).

The raw data were loaded into Seurat using the Load10X_Spatial() function, and then read by a Spaceranger to return an object containing both gene expression data and related images of tissue sections. ST data were qualitatively assessed based on several parameters, including the total spots and average UMI genes per spot. Genes less than 200 per spot or those expressed in fewer than three spots were omitted. The logVMR function was used to normalise the data across spots. PCA was used to perform dimensionality reduction and clustering. The AddModuleScore() function was used to calculate the cell signature score for each ST spot. The SPOTlight approach, a computational tool that integrates ST with scRNA-seq data, was used for deconvolution to map the cell types and states within complex tissues [20]. The SpatialFeaturePlot() function can overlay molecular data on top of tissue histology.

### Statistical analyses

All statistical analyses were performed using SPSS software (version 26.0), GraphPad Prism software (version 9.0), and R (version 4.2.1). The significance of the differences was determined using the Mann–Whitney *U* test or Kruskal–Wallis test as needed. Dunn’s adjustment was applied for multiple comparisons of immune cell characteristics and TLS location (TC, IM, and N) [21]. Cumulative survival time was estimated using the Kaplan–Meier method, and significance was assessed using the log-rank test. Immune-related overall survival (irOS) was defined as the interval between the initiation of immunotherapy for advanced GC and death of any cause or the end of follow-up. Immune-related progression-free survival (irPFS) was defined as duration from the initiation immunotherapy to the day of disease progression, death or the last follow-up, whichever came first. Overall survival (OS) is the interval between diagnosis and death of any cause or the last follow-up. Progression-free survival (PFS) was calculated as the time from diagnosis to disease progression or death from any cause. Prognostic factors were analysed using LASSO regression analyses and ten-fold cross-validation. The predictive performance of the risk model was assessed using the receiver operating characteristic curve and the time-dependent area under the curve. Statistical significance was set at a two-tailed *P*-value less than 0.05.

## Results

### Presence of TC-TLS is associated with better survival

To evaluate the correlation between the presence of TLS and prognosis in GC, TLS were initially assessed based on clinicopathological characteristics analysed using H&E staining of surgical samples from 59 patients with GC who underwent anti-PD-1/PD-L1 therapy (Fig. 1A). Every patient was enrolled in this study between 1 January 2016 and 1 June 2020. As presented in Table 1, 55% of the GC samples contained intratumoural TLS in the TC. Tumour differentiation was poor in 27 cases, moderate in 22, and high in seven. Twelve (20.3%) tumours were HER2-positive. Mismatch repair deficiency was observed in seven cases (11.9%), and a total of four (6.8%) tumours were positive for EBER ISH. According to PD-L1 expression, a combined positive score (CPS) ≥ 1 was found in 32 cases (54.2%), while 18 patients (30.5%) had a CPS ≥ 5. Importantly, our analysis revealed no association between TLS and age, sex, stage, location, differentiation, Lauren classification, HER2 status, EBV status, PD-L1 CPS score, or MMR status. The existence of intratumoural TLS in TC was significantly associated with better irOS (30.3 months versus not reached, Log-rank *P* = 0.049, HR = 0.37) in GC (Fig. 1C). A similar trend was observed in irPFS (Additional file 1: Fig. S1B), suggesting that TLS may be a favourable prognostic indicator in GC.

### CD8^+^PD-1^+^ cell subtype is enriched in TC-TLS and associated with better prognosis

We employed a multiplex IHC assay to evaluate tumour microenvironment, staining serial tissue sections with five different panels (Fig. 1B). To understand the differences in the internal and external TLS microenvironment in the TC region, we analysed the densities of immune cell subsets in the TLS, tumour, and stromal regions (Fig. 2A). A significant increase in the overall density of CD20^+^ and CD21^+^ B cells, CD3^+^, CD8^+^ and CD4^+^ T cells was observed in TC-TLS compared with that in the stroma and tumour tissues (Additional file 1: Fig. S2A). Furthermore, we conducted a detailed exploration of the distribution of each TIIC type. Within the TC-TLS, there was a greater abundance of various T cell subsets, including CD8^+^PD-1^+^, CD8^+^PD-1^+^TIM-3^−^, CD8^+^LAG-3^−^PD-1^+^TIM-3^−^, CD8^+^TIM-3-, CD8^+^LAG-3^−^TIM-3^−^, CD4^+^FoxP3^−^, CD4^+^CTLA-4^−^, and CD4^+^PD-L1^+^ T cells, compared with that in the tumour and stromal regions. Additionally, FAP^+^SMA^+^ fibroblast populations were found to be more specific to the stromal area (Fig. 2D).

We found that increased levels of tumour-infiltrating T cell subsets, specifically CD8^+^PD-1^+^, CD8^+^PD-1^+^TIM-3^−^, and CD8^+^LAG-3^−^PD-1^+^TIM-3^−^ T cells, were significantly correlated with improved OS (*P* = 0.032, 0.032, and 0.023 respectively). PFS exhibited a similar pattern (Additional file 1: Fig. S1C). These findings underscore the importance of the immune microenvironment within TLS and reveal that the presence of specific T cell subsets within TLS can potentially affect patient outcomes.

### The TLS and non-TLS zones exhibit distinct immune microenvironments within the tumour IM and peri-cancerous normal area

We further examined the localisation of TLS in the IM and normal tissues (Fig. 2B, C). Significantly higher densities of CD20^+^ and CD21^+^ B cells, as well as CD3^+^, CD8^+^, and CD4^+^ T cells, were observed within TLS regions than in adjacent non-TLS tissues, indicating a predominance of T and B cells within the TLS (Additional file 1: Fig. S2B, C).

We quantified the densities of TIICs within the IM by comparing the densities in the IM-TLS and IM-non-TLS zones. CD8^+^LAG-3^−^TIM-3^−^, CD8^+^PD-1^−^, CD8^+^LAG-3^−^PD-1^−^TIM-3^−^, and CD4^+^CTLA-4^−^FoxP3^−^PD-L1^−^ T cells concentrated in the IM-TLS areas and showed reduced in the IM-non-TLS areas. In contrast, there was a notable increase in the numbers of CD8^+^LAG-3^+^PD-1^+^TIM-3^+^ T cells, TIM-3^+^ cells, CD68^+^CD86^+^STING^+^ macrophages, and FAP^+^ fibroblasts in the IM-non-TLS zones (Fig. 2E).

Furthermore, a detailed analysis of normal tissues revealed that populations of CD8^+^PD-1^+^, CD8^+^LAG-3^+^PD-1^+^TIM-3^+^, CD4^+^CTLA-4^+^, and CD4^+^PD-L1^+^ T cells were predominantly found in the normal tissue (N)-TLS areas. In contrast, significantly higher densities of CD68^+^CD86^+^ and CD163^+^CD86^+^STING^+^ macrophages, CD66b^+^ neutrophils, and FAP^+^SMA^+^ fibroblasts were predominantly localised to the N-non-TLS compared to those in the N-TLS, showing marked specificity (Fig. 2F). The varying levels of TIICs across different locations underscore the heterogeneous distribution of TIICs in GC.

### The spatial distribution of immune cells in TLS within the TC and its prognostic value

**Fig. 3:**
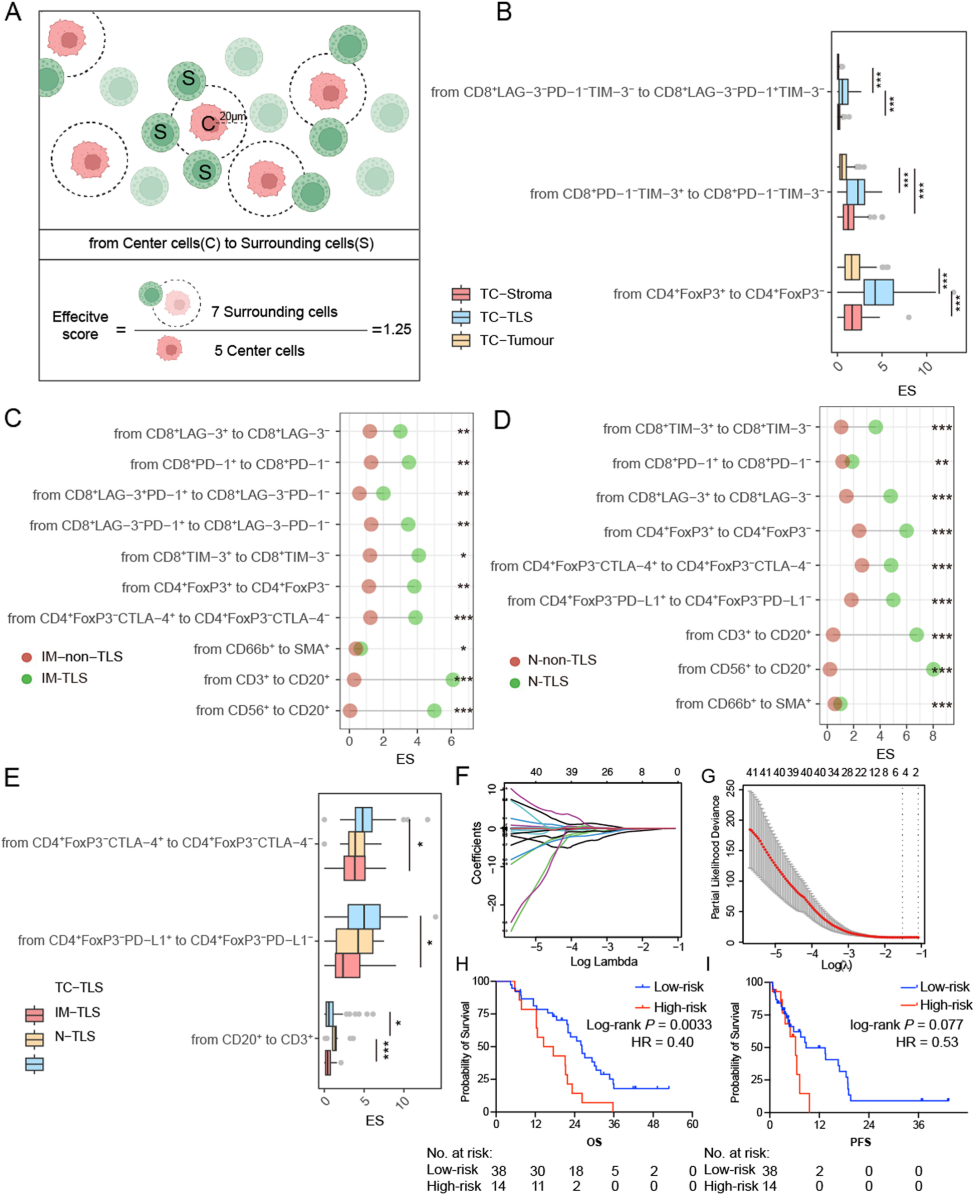
Distinct spatial distribution of immune cells across TLS in diverse locations. **A** Illustration of the distance analysis involving centre and surrounding cells. Red dots: centre cells; green dots: surrounding cells. The white translucent circle represents the radius. Effective score = number of paired surrounding cells and tumour cells/number of centre cells. Scale bar: 100 μm. **B** Comparison of the effective scores between TC-Tumour, TC-TLS, and TC-Stroma regions. TC-Tumour, tumour in the tumour core; TC-TLS: TLS in the tumour core; TC-Stroma, stroma regions in the tumour core. Each point represents one patient. *p < 0.05, **p < 0.01, ***p < 0.001 and not significant (ns). **C** Comparison of the effective scores between IM-non-TLS and IM-TLS. Red dots: IM-non-TLS; green dots: IM-TLS. IM-TLS, TLS in the invasion margin; IM-Non-TLS, invasion margin excluding TLS. *p < 0.05, **p < 0.01, ***p < 0.001 and not significant (ns). **D** Comparison of the effective scores between N-non-TLS and N-TLS. Red dots: N-non-TLS; Green dots: N-TLS. N-TLS, TLS in the normal tissue; N-Non-TLS, normal tissue excluding TLS. *p < 0.05, **p < 0.01, ***p < 0.001 and not significant (ns). **E** Comparison of the effective scores between TC-TLS, IM-TLS, and N-TLS. *p < 0.05, **p < 0.01, ***p < 0.001 and not significant (ns). **F**, **G** Five robust density and spatial score (DSS) were selected by the LASSO Cox regression model. **H**, **I** Kaplan–Meier curve showing the overall survival (OS) and progression-free survival (PFS) rate differences between high and low-DSS groups. TC-TLS were identified in a total of 52 GC patients

In our endeavour to precisely characterise the spatial organisation of individual cells in GC, we used high-resolution cell-mapping techniques. For a detailed examination of the localisation patterns between the two distinct cell types, we employed the bioinformatics tool to calculate the nucleus-to-nucleus distances. An “effective score” was developed to consider the proximity and quantity of these cells. This metric quantifies the interactions between the central and surrounding cells as depicted in Fig. 3A. A preselected radius of 20 μm was employed to identify immune cell likely to interact effectively with central cells. This radius was consistent with prior studies in multiple gastrointestinal tumour types [22–26]. Therefore, this effective score was calculated by dividing the number of paired surrounding cells by the total number of central cells surrounded by cells across the entire slide, to effectively preserve the observed spatial variation. A high effective score indicated more pronounced cell-by-cell encirclement.

In the TC-TLS, the effective scores of CD8^+^LAG-3^−^PD-1^−^TIM-3^−^ T cells (surrounding cells: CD8^+^LAG-3^−^PD-1^+^TIM-3^−^), CD8^+^PD-1^−^TIM-3^+^ T cells (surrounding cells: CD8^+^PD-1^−^TIM-3^−^), and CD4^+^FoxP3^+^ T cells (surrounding cells: CD4^+^FoxP3^−^) were notably higher than those in the tumour and the stroma (Fig. 3B). Notably, patients with higher effective scores of CD8^+^LAG-3^−^PD-1^−^TIM-3^−^ T cells (surrounding cells: CD8^+^LAG-3^−^PD-1^+^TIM-3^−^) and CD8^+^PD-1^−^TIM-3^+^ T cells (surrounding cells: CD8^+^PD-1^−^TIM-3^−^) in the TC-TLS showed significantly longer OS than those with lower effective scores (*P* = 0.012). Additionally, patients with higher effective scores of CD8^+^PD-1^−^TIM-3^+^ T cells (surrounding cells: CD8^+^PD-1^−^TIM-3^−^) showed significantly longer PFS than those with lower effective scores (*P* = 0.036). Conversely, patients with higher effective scores of CD4^+^FoxP3^+^ T cells (surrounding cells: CD4^+^FoxP3^−^) were associated with inferior PFS (*P* = 0.016) (Additional file 1: Fig. S3A). This comprehensive analysis highlights the crucial role of spatial cellular interactions in GC, which significantly affect patient outcomes.

### The spatial distribution of immune cells within TLS is more closely associated in IM and normal zones

To delve deeper into the spatial distribution of immune cells in the IM and normal tissue areas, we compared the internal and external sections of TLS in these zones. The effective scores of CD8^+^LAG-3^+^ T cells (surrounding cells: CD8^+^LAG-3^−^), CD8^+^PD-1^+^ T cells (surrounding cells: CD8^+^PD-1^−^), CD8^+^TIM-3^+^ T cells (surrounding cells: CD8^+^TIM-3^−^), and CD4^+^FoxP3^−^CTLA-4^+^ T cells (surrounding cells: CD4^+^FoxP3^−^CTLA-4^−^) were internally higher in TLS (Fig. 3C, D). The higher effective scores of CD3^+^ T cells (surrounding cells: CD20^+^ B cells) an CD56^+^ natural killer (NK) cells (surrounding cells: CD20^+^ B cells) were observed inside TLS compared with those outside TLS, indicating a more intense surrounding of T cells by B cells within the TLS. Especially, the effective score of CD4^+^FoxP3^+^ T cells (surrounding cells: CD4^+^FoxP3^−^) was higher in the IM-TLS compared with IM-non-TLS. Additionally, intra-TLS heterogeneity was explored across the TC, IM, and normal tissues. The effective score of CD20^+^ T cells (surrounding cells: CD3^+^) was the highest in the IM, followed by the normal tissue and TC. The effective scores of CD4^+^FoxP3^−^CTLA-4^+^ T cells (surrounding cells: CD4^+^FoxP3^−^CTLA-4^−^) and CD4^+^FoxP3^−^PD-L1^+^ T cells (surrounding cells: CD4^+^FoxP3^−^PD-L1^−^) were highest in the normal tissues (Fig. 3E). Notably, these findings highlight that while immune cells within the TLS are in close proximity, they also exhibit structural differences across different regions.

### Constructing a risk score to predict the prognosis of GC patients

Considering that the density and spatial characteristics of immune cells in TC-TLS are closely related to prognosis, we recruited 143 TIIC characteristics into the Least Absolute Shrinkage and Selection Operator (LASSO) Cox regression model to screen robust prognosticators. Eventually, five characteristics were selected to build a density and spatial score (DSS) risk model (Fig. 3F, G). Then the risk score per patient was determined as: DSS = (− 0.0027 × the density of CD8^+^LAG-3^+^) + (− 0.0003 × the density of CD4^+^FoxP3^+^CTLA-4^+^) + (− 0.0004 × the density of CD4^+^CTLA4^+^PD-L1^+^) + (− 0.1978 × the effective score of CD8^+^LAG-3^−^PD-1^−^TIM-3^−^ (surrounding cells: CD8^+^LAG-3^−^PD-1^−^TIM-3^+^) + (− 0.1214 × the effective score of CD8^+^LAG-3^−^PD-1^+^TIM-3^−^ (surrounding cells: CD8^+^LAG-3^−^PD-1^+^TIM-3^+^). Based on the optimal cutoff value, the patients were subsequently divided into high- and low-DSS groups. Low-DSS groups displayed a significantly longer OS than those with a high DSS (26.20 months versus 16.02 months, Log-rank *P* = 0.0033, HR = 0.40). PFS displayed a similar trend (Fig. 3H, I).

### The intercellular communication is associated with antigen presentation and cancer cell pyroptosis in the TLS^+^ environments

**Fig. 4:**
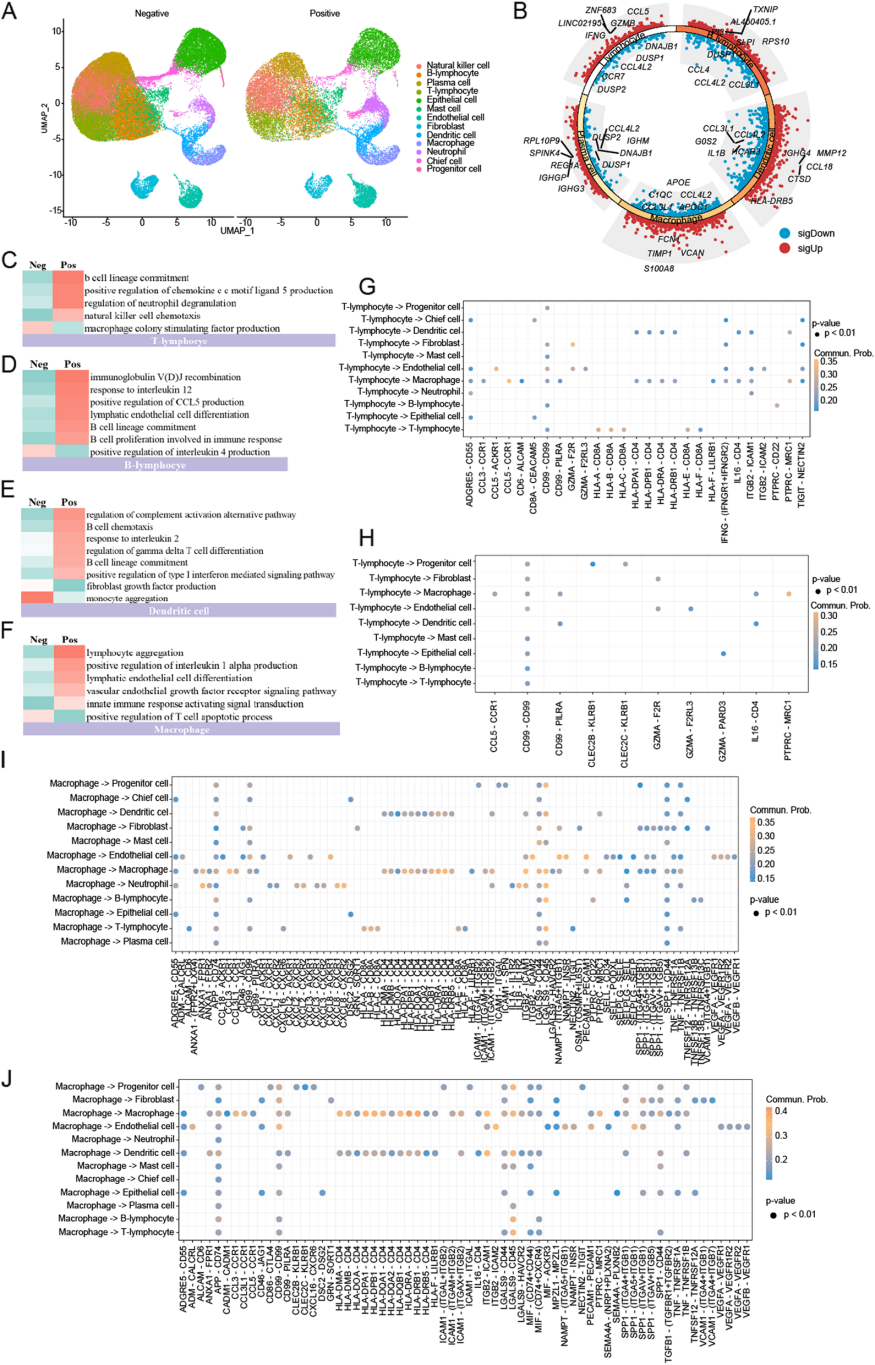
Gene expression and interaction network differences in TLS-positive and TLS-negative microenvironments using single-cell RNA data. **A** UMAP plots demonstrated the identified cell lineages from 12 primary GC tissues and paired TLS^−^ (Left) and TLS^+^ (Right). **B** Volcano plot displayed the differentially expressed genes (DEGs) in these annotated subclusters. **C**–**F** Gene set variation analyses compared pathway activity of T cells (**C**), B cells (**D**), dendritic cells (**E**), and macrophages (**F**) among TLS^+^ and TLS^−^ group by enrichment scores. **G**, **H** Depiction of interaction probabilities with T cells as signal transmitters and other cell types as signal receivers mediated by ligand–receptor pairs in TLS^+^ (**G**) and TLS^−^ (**H**) group. **I**, **J** Depiction of interaction probabilities with macrophages as signal transmitters and other cell types as signal receivers mediated by ligand–receptor pairs in TLS^+^ (**I**) and TLS^−^ (**J**) group. GC, gastric cancer

To profile the molecular and functional changes in cells and their roles in modulating cancer development, scRNA-seq was conducted on samples in the presence and absence of the TLS (Fig. 4A). It contained four samples exhibiting TLS and eight samples lacking TLS (TLS^+^ and TLS^−^ groups, respectively). After quality control and data preprocessing, all the cells from the 12 samples were categorised into 13 clusters based on their canonical markers: T, B, NK, plasma, dendritic, macrophage, neutrophil, endothelial, fibroblast, chief, progenitor, mast cells, and ECs. Compared with TLS^−^ group, T cells expressed high levels of GZMB and IFNG in TLS^+^ group (Fig. 4B). GSVA showed that DEGs of T cells between TLS^+^ and TLS^−^ groups were enriched into B cell lineage commitment and NK cell chemotaxis (Fig. 4C). Furthermore, the DEGs of B cells in the two groups were enriched in response to interleukin 12 (Fig. 4D). The interleukin 2 pathway was enriched in dendritic cell subclusters (Fig. 4E) and the positive regulation of interleukin 1-α production pathway was enriched in macrophage subclusters (Fig. 4F).

We further assessed the crosstalk strength mediated by ligand-receptor pairs between T cells, macrophages, and other cell types. In the TLS-positive group, T cells may promote pyroptosis via the GZMA-F2R/F2RL3 axis through interaction with endothelial cells. T cells also recruited macrophages via the CCL3/5-CCR1 and CD6-ALCAM axes (Fig. 4G, H ). Macrophages mainly communicated with T cells (HLA − A/B/C − CD8A) by antigen presentation process (Fig. 4I, J). Collectively, these findings suggested a marked enhancement in the interactions between T cells and other cell types within the TLS-positive TME, particularly between T cells and macrophages, as reflected by the increased expression of macrophage ligands by T cells.

### The enhanced interaction of tumour cells in the TLS-deficient TME is mostly related to angiogenesis and tumour immunosuppression

**Fig. 5:**
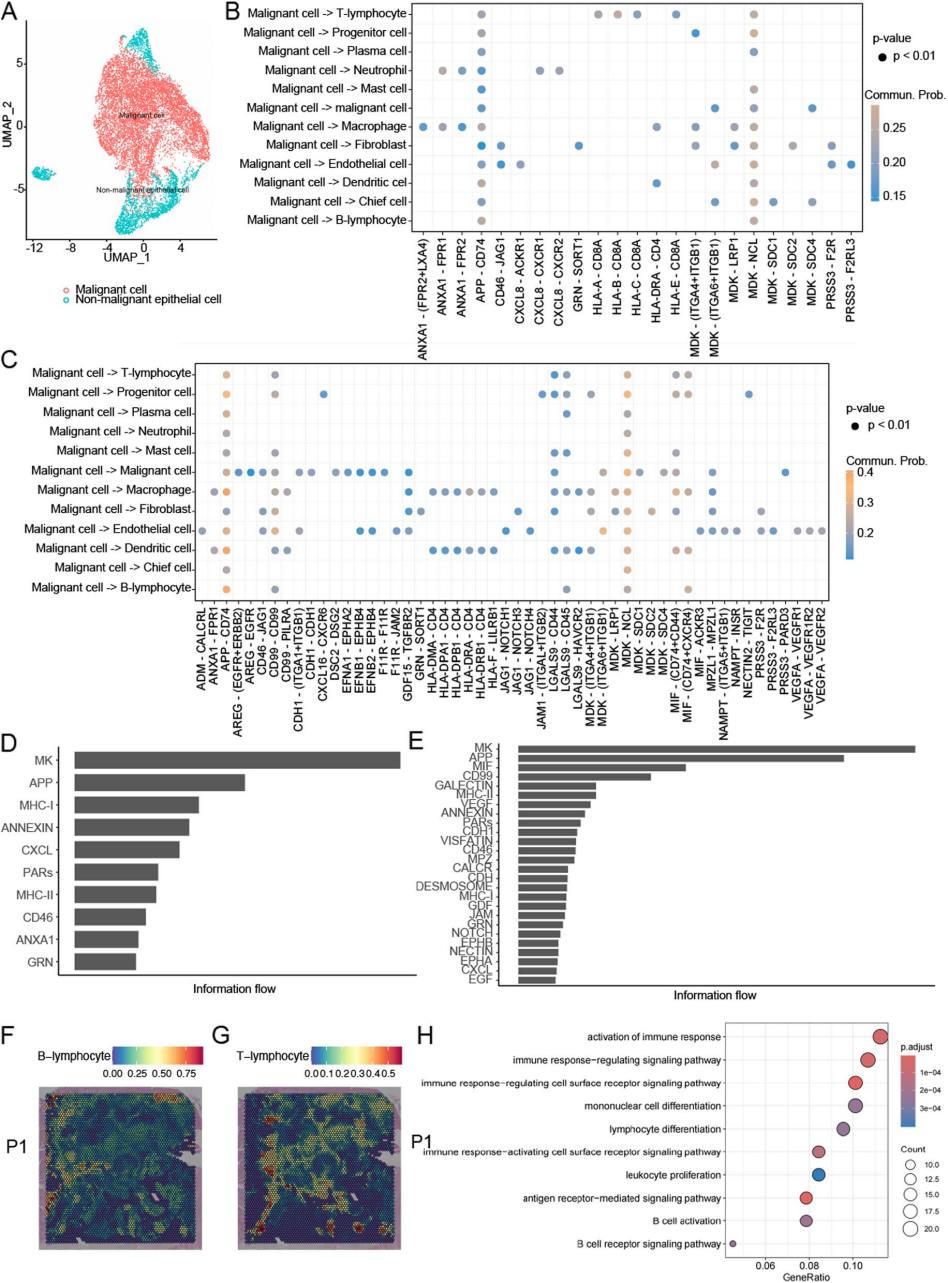
The enhanced interaction of tumour cells in the TLS-deficient microenvironment is largely related to immunosuppression. **A** UMAP plots showed the two annotated clusters including malignant cells and non-malignant ECs. **B**, **C** Depiction of interaction probabilities with tumour cells as signal transmitters and other cell types as signal receivers mediated by ligand–receptor pairs in TLS^+^ (**B**) and TLS^−^ (**C**) group. **D**, **E** Comparison of the overall information flow of each signal pathway with tumour cell as signal transmitters between TLS^+^ (**D**) and TLS^−^ (**E**) group. **F**, **G** Spatial feature plots of signature score of B cells (**F**), and T cells (**G**) in P1. **H** Gene ontology (GO) analysis showing enriched biological process terms of differentially expressed genes (DEGs) in TLS than tumour in P1. TLS, tertiary lymphoid structure; UMAP, Uniform Manifold Approximate and Projection

To further explore the impact of tumour cells on TLS, we conducted a detailed comparison of malignant cell-to-cell communication between the TLS-positive and TLS-negative groups. The annotation of tumour cells for GC was confirmed by the “inferCNV” package (Fig. 5A). An inferCNV-clustered heat map was created, which corresponded to the normalised expression values of immune cells plotted in the top panel and ECs in the bottom panel (Additional file 1: Fig. S4A). In the resultant CNV heatmap, the regions of CNV amplification are depicted in red and regions of CNV deletion are depicted in blue. Darker colours indicate more pronounced CNV variants. The cluster labelled “malignant cell” showed high expression levels of the representative identified marker genes (Additional file 1: Fig. S4B). In TLS-positive environments, malignant cells induced T-cell activation through the HLA − A/B/C − CD8A interaction (Fig. 5B). Conversely, in the absence of TLS, malignant cells recruited endothelial cells via GDF15-TGFBR2, thereby exerting a key immunosuppressive role. Additionally, the TLS-negative group exhibited significantly enhanced interactions via ligand-receptor pairs, such as LGALS9-CD44 (Galectin9-CD44), MDK-NCL, and MIF-(CD74 + CD44) (Fig. 5C). We then evaluated the signalling pathway activity between the TLS-positive and -negative groups. The interactions of MIF, GALECTIN, and VEGF were significantly higher in the TLS-negative group, than in the TLS-positive group (Fig. 5D, E). Collectively, cell–cell communication analysis revealed a suppressive immune response and induction of angiogenesis and tumour invasiveness in malignant cells in the TLS-negative group.

### ST analysis revealed that TLS associates with the activation of immune response

To analyse the impact of the TLS on the TME architecture at a spatial level and comprehensively investigate the spatial heterogeneity of GC, two primary tumours were collected using FFPE sections to perform Visium ST sequencing using 10 × Genomics. The overall organisation of each tumour and the presence and localisation of TLS were annotated by a trained pathologist (H.Y.J.) after H&E staining. The tissue was segmented into tumour and TLS areas (Additional file 1: Fig. S5A). The spatial characteristics of the major cell populations were delineated by mapping the scRNA-seq data onto ST slides using the SPOTlight algorithm. UMAP analysis identified 15 clusters in both the P1 and P2 samples. Notably, Cluster 13 of P1 and Cluster 1 of P2, both characterised by high expression levels of immune markers, such as CD3G, IGHG1, IGHG3, FCRL5, and FCRL2, contained TLS (Additional file 1: Fig. S5B). Cluster 2 of P1 and cluster 7 of P2 were identified as tumours. IGHG1 and IGHG3 are immunoglobulin markers. FCRL5 is expressed in plasma cells and promotes B cell proliferation after antigen exposure. These findings were further validated by detailed histomorphological analyses conducted by an experienced pathologist.

This analysis underscored the pronounced colocalisation of T and B cells within the TLS areas, suggesting a synergistic interaction within these structures (Fig. 5F, G, Additional file 1: Fig. S5C). Subsequent investigations into the biological processes distinguishing the TLS from the tumour region revealed that the DEGs between these regions predominantly fell into categories associated with the activation of the immune response, immune response-regulating signalling pathways, and B cell activation, supporting the scRNA-seq findings (Fig. 5H, Additional file 1: Fig. S5D).

This comprehensive analysis suggests that the formation of TLS within GC tumours might be a crucial factor in fostering a TME characterised by high levels of immune infiltration and an active immune response.

## Discussion

In this study, through morphological examination of tissue slides from surgically resected GC tumours, we showed that TC-TLS correlates with a better prognosis in patients undergoing immunotherapy. To our knowledge, this is the first time the internal and external TLS have been explored, especially the prognosis-related features in internal TLS. We used the density and spatial characteristics of TIIC to portray the TLS-associated TME and constructed a prognostic model which could be a meaningful tool for predicting the prognosis of GC.

The TLS contributes to a beneficial microenvironment that supports the aggregation of immune cells and the humoral responses [8, 27]. Memory T cells, B cells, long-lived plasma cells, and other immune-related cells can establish a lasting immunological foundation, suggesting that TLSs either directly contribute to the immunotherapy response or report on a tumour microenvironment conducive to immunotherapy [28, 29]. In our study, TLS in TC notably exhibited a higher concentration of CD8^+^PD-1^+^ cells within CD8^+^ T cells than in peripheral TLS areas. The enrichment of various CD8^+^ T cell subsets, particularly those expressing PD-1, not expressing TIM-3, and expressing LAG-3, was strongly linked to improvements in OS, consistent with prior research that linked CD8^+^PD-1^+^ T cell infiltration with therapeutic responses in proficient mismatch repair colon cancers [30]. Thommen et al. further illuminated this observation by demonstrating that PD-1^+^CD8^+^ T cells produce CXCL13, a key mediator for recruiting immune cells to the TLS, thus enhancing their prognostic significance [31].

Spatial organisation analysis also underscored the importance of the precise arrangement of immune cells within the TLS. Our “effective score” metric, reflecting the proximity between central and surrounding cells, highlighted the significant positioning of CD8^+^PD-1^+^ T cells in these structures. However, a high effective score of CD4^+^FoxP3^+^ T cells (surrounded by CD4^+^FoxP3^−^ cells) was associated with poorer PFS. Hirschhorn et al. [32] suggested that regulatory CD4^+^T (Treg) cells within tumour-associated TLS might dampen antitumour responses, shifting towards a more immunostimulatory environment following Treg cell depletion. These results underscore the dual nature of immune responses within the TLS, particularly highlighting the nuanced and potent immunosuppressive role of Treg cells in shaping tumour adaptive immunity and sparking further enquiry into the mechanisms by which Treg cells influence the antitumour immune landscape.

The significance of TLS located in the IM and normal peritumoural areas in GC has been less explored, with limited data on their prognostic value. The impact of peritumoural TLS on prognosis appears to vary by cancer type; for instance, in hepatocellular carcinoma and breast cancer, the presence of peritumoural TLS has been associated with poorer OS [7, 33]. Therefore, a more comprehensive analysis of the assessment of TLS characteristics depending on location is required. In our analysis, we identified three distinct localisations of TLS in GC: TC, IM, and normal adjacent tissues, each showing a unique cellular composition. Notably, TLS at the IM were enriched with non-exhausted CD8^+^ T cells (CD8^+^PD-1^−^LAG-3^−^TIM-3^−^), contrasting with those in normal tissue, which were more likely to include exhausted CD8^+^ T cells (CD8^+^PD-1^+^LAG-3^+^TIM-3^+^) and M2 macrophages expressed STING (CD68^+^CD86^+^STING^+^). This variation highlights the diverse immune profiles across tumour regions and emphasises the heterogeneity of TIICs. Upregulation of PD-1 and LAG-3 is indicative of T cell exhaustion, leading to increased tolerance to self and tumour antigens and facilitating tumour immune evasion [34, 35]*.* The dual therapy with anti-LAG-3 and anti-PD-1 antibodies counteracts these effects and potentially eradicate tumours [36]. Additionally, the prominent expression of TIM-3 in the tumour environment supports tumour immune escape by promoting the apoptosis of effective T cells, enhancing the proliferation of regulatory and inhibitory T cells, and inhibiting the efficacy of NK and dendritic cells [37, 38]. The high expression of immune exhaustion genes, including *PD-L1*, *LAG3*, and *T-bet*, in CD8^+^ T cells has been linked to reduced OS in colorectal cancer [39]. M2 macrophages are abundant in TLS within normal tissues and can facilitate tumour growth by inducing immune suppression [40]. M2 macrophages secrete CHI3L1 to promote GC metastasis [41]. Our findings suggest that TLS can serve both tumour-promoting and tumour-suppressing functions depending on the specific immune cell types they contain. Specifically, our results demonstrate that peritumoural TLS contribute to an immunosuppressive milieu that favours tumour growth, shedding light on the negative prognostic association observed with peritumoural TLS in some cancers.

Further spatial analysis indicated that B cells surrounded by T cells achieved the highest effective score within the TLS at the IM and the lowest in the TC. Wu [42] found that the IM, a 500-µm-wide zone centred around the tumour border, is a site of pronounced immunosuppression and metabolic reprogramming that potentially facilitates tumour progression. The IM is considered a complex and dynamic local ecosystem crucial for tumourigenesis, where immune and tumour cells are particularly active. This highlights the importance of focusing on the IM in our study. Effective antitumour immunity requires TLSs to have a spatial structural basis; however, the degree to which T cells surround B cells may not be a crucial factor. Our study found that regardless of the TLS location, the spatial proximity of immune cells within the TLS was stronger than that outside the TLS, indicating an increased likelihood of cell–cell contact. The formation of the TLS involves lymphocytes interacting with stromal cells, leading to the secretion of chemokines, such as CXCL13 and IL7, which attract more immune cells to the site [8]. Most TLS lack a complete fibrous capsule, allowing immune cells within the TLS to contact the surrounding tissues directly. This direct contact facilitates faster antigen recognition and generation of adaptive immune responses [43].

Notably, in our study, scRNA-seq revealed that cell-to-cell interactions were more robust in the presence of TLS in GC. We identified specific ligand-receptor pairs that illuminate a unique pattern of intercellular communication in these microenvironments. We observed that the interaction between HLA-A/B/C and CD8A might drive immune responses, which are crucial for cellular defence mechanisms [44]. The bond between GZMA released by cytotoxic cells and F2R on tumour cells triggers tumour suppression and T cell-mediated destruction by activating the JAK2/STAT1 pathway [45]. GZMA-mediated activation of GSDMB induces extensive pyroptosis in target cells, potentially bolstering anti-tumour immunity [46]. Additionally, the relationship exhibited by CCL3/5 and CCR1 was found to correspond with pro-inflammatory macrophage polarisation, which in turn facilitates the phagocytosis of cancer cells [47, 48]. The CD6/ALCAM pathway is essential for T cell activation and proliferation [49]. Additionally, there was an upregulation in the functions associated with the recruitment of B cells and chemotaxis of NK cells. Cellular interactions occur more frequently in environments containing TLS, contributing to a dynamically active immune microenvironment.

Conversely, in TLS-deficient microenvironments, the relationship between GDF15 and TGFBR2 is associated with tumour invasion and subsequent metastasis. Tumour cells facilitate interactions with immune cells via MIF- and galectin-dependent pathways. The cytokine MIF facilitates the proliferation of myeloid-derived suppressor cells [50] and encourages M2 macrophage polarisation [51]. Galectins are crucial in cancer progression and have attracted considerable interest owing to their prognostic and therapeutic implications [52, 53].

This study had certain limitations. First, the cohort to evaluate the predictive value of TC-TLS is small and all tumour samples were obtained from a single institution. However, we classified 86 immune cell subtypes according to the positivity and relative intensity of twenty-two markers in the five m-IHC panels to identify the detailed TME characteristics for each patient. Second, the retrospective design of the study reduced the clinical significance of our findings. Therefore, future prospective cohort studies are necessary to validate our proposed hypotheses. In addition, the mechanism by which TLS localisation heterogeneity affects abnormal GC TME phenotypes needs to be further explored. Exploration using an in situ xenografted tumour model or a patient-derived tumour organoid/patient-derived tumour xenograft model to induce TLS might help direct future research.

## Conclusions

Our results outlined the spatial heterogeneity of TLS characteristics and presented a novel risk model for predicting survival in GC patients. We also identified the critical role of frequent cellular communication within the TLS in shaping an active immune microenvironment, revealing the critical role of various cells in determining the anti- or pro-tumour abilities of TLS and providing potential strategies to enhance immunotherapy response rates.

## Supplementary Information

Additional file 1: Fig. S1. The presence of TC-TLS and CD8^+^PD-1^+^ T cells within TC-TLS are associated with a favourable prognosis. (A) Selection of the regions of interest (ROIs) in representative images of haematoxylin and eosin (H&E)-stained formalin-fixed paraffin-embedded tissues. TC, tumour core; IM, invasion margin; N, normal tissue. Scale bar: 2 mm. (B) Kaplan–Meier survival curves for irPFS of patients with and without TLS in the tumour core. *P*-values are two sided. (C) PFS and OS of patients with GC based on the density of tumour-infiltrating immune cells (TIICs). The individual TIICs were divided into high (>half of the patients; red line) or low density (≤half of patients; blue line). The log-rank (Mantel–Cox) test was used. A two-sided *P* < 0.05 was considered statistically significant. GC, gastric cancer; TLS, tertiary lymphoid structure; irPFS, immune-related progression-free survival; PFS, progression-free survival; OS, overall survival. Fig. S2. The immune composition inside and outside TLS in different regions. (A–C). The density of main cell types of tumour core (A), invasion margin (B) and normal tissue (C). Box and whiskers represent mean ± 10–90 percentile. Three groups were compared: Kruskal–Wallis test with Dunn’s multiple comparison test. Two groups were compared: Wilcox test. Each point represents one patient. *p < 0.05, **p < 0.01, ***p < 0.001 and not significant (ns). TLS, tertiary lymphoid structure; TC-TLS, TLS in the tumour core; TC-stroma, stromal regions in the tumour core; IM-TLS, TLS in the invasion margin; IM-Non-TLS, invasion margin excluding TLS; N-TLS, TLS in normal tissue; N-Non-TLS, normal tissue excluding TLS. TLS, tertiary lymphoid structure. Fig. S3. Survival analysis based on the effective scores. (A) PFS and OS of patients with GC based on the effective scores of tumour-infiltrating immune cells (TIICs). The effective scores were divided into high (>half of the patients; red line) or low density (≤half of patients; blue line). Log-rank (Mantel–Cox) test was used. A two-sided *p* < 0.05 was considered statistically significant. PFS, progression-free survival; OS, overall survival. Fig. S4. Malignant epithelial cells identified by CNV levels. (A) The hierarchical heatmap showing large-scale CNVs in tumour lesions. (B) Violin plots for marker genes of cancer cells (CEACAM5, KRT23, CLDN3, and CLDN4). Fig. S5. Spatial transcriptomic suggests a “hot” TME in GC. (A) Left to right: Haematoxylin and eosin (H&E) stained tissues which are delineated into tumour (red) and TLS (blue), UMAP of the cluster identities, spatial distribution of the clusters in two cases of GC undergoing spatial transcriptomic sequencing. (B) Volcano plot displayed the differentially expressed genes (DEGs) in TLS than the tumour regions. (C) Spatial feature plots of signature score of B cells, and T cells in P2. (D) Gene ontology (GO) analysis showing enriched biological process terms of DEGs in TLS than the tumour regions in P2. GC, gastric cancer; TLS, tertiary lymphoid structure; UMAP, Uniform Manifold Approximation and Projection.

Additional file 2: Table S1. Summary of recurrence and distant metastasis in stage I–III patients (*N*=16). Table S2. The baseline characteristics of 110 gastric adenocarcinoma patients. Table S3. The baseline characteristics of gastric adenocarcinoma patients in spatial transcriptomic cohort (*N*=2). Table S4. The baseline characteristics of gastric adenocarcinoma patients in single-cell sequencing cohort (*N*=12). Table S5. Summary of primary antibodies and secondary antibody used for multiplex immunohistochemistry.

## Data Availability

No datasets were generated or analysed during the current study.

## Abbreviations

GC: Gastric cancer
TLS: Tertiary lymphoid structures
TME: Tumour microenvironment
H&E: Haematoxylin and eosin
TC: Tumour core
IM: Invasion margin
N: Surrounding peri-cancerous normal
TIIC: Tumour-infiltrating immune cells
scRNA-seq: Single-cell RNA sequencing
ST: Spatial transcriptomic
CNV: Copy number variation
DEGs: Differentially expressed genes
H&E: Haematoxylin-eosin
irPFS: Immune-related progression-free survival
irOS: Immune-related overall survival
LGALS9: Galectin-9
NK: Natural killer cell
PBS: Phosphate-buffered saline
PCA: Principal component analysis
m-IHC: Multiplex immunohistochemistry
GSVA: Gene set variation analysis
TGF-β: Transforming growth factor-β
UMI: Unique molecular identifiers
ECs: Epithelial cells
FFPE: Formalin-fixed and paraffin-embedded
ROIs: Regions of interest
UMAP: Uniform Manifold Approximation and Projection
DEGs: Differentially expressed genes
LASSO: Least Absolute Shrinkage and Selection Operator
HER2: Human epidermal growth factor receptor 2
CPS: Combined positive score
GEJ: Gastro-oesophageal junction
EGFR: Epidermal growth factor receptor
TSA: Tyramine signal amplification
Treg: Regulatory CD4^+^T
