# Supplementary material for: Immune microenvironment spatial landscapes of tertiary lymphoid structures in gastric cancer
Source: BMC Med. 2025 Feb 4;23:59. doi: 10.1186/s12916-025-03889-3 (PMC11792408; doi:10.1186/s12916-025-03889-3)
Supplement: Supplementary file 1 — Additional file 1: Fig. S1. The presence of TC-TLS and CD8+PD-1+ T cells within TC-TLS are associated with a favourable prognosis. (A) Selection of the regions of interest (ROIs) in representative images of haematoxylin and eosin (H&E)-stained formalin-fixed paraffin-embedded tissues. TC, tumour core; IM, invasion margin; N, normal tissue. Scale bar: 2 mm. (B) Kaplan–Meier survival curves for irPFS of patients with and without TLS in the tumour core. P-values are two sided. (C) PFS and OS of patients with GC based on the density of tumour-infiltrating immune cells (TIICs). The individual TIICs were divided into high (>half of the patients; red line) or low density (≤half of patients; blue line). The log-rank (Mantel–Cox) test was used. A two-sided P < 0.05 was considered statistically significant. GC, gastric cancer; TLS, tertiary lymphoid structure; irPFS, immune-related progression-free survival; PFS, progression-free survival; OS, overall survival. Fig. S2. The immune composition inside and outside TLS in different regions. (A–C). The density of main cell types of tumour core (A), invasion margin (B) and normal tissue (C). Box and whiskers represent mean ± 10–90 percentile. Three groups were compared: Kruskal–Wallis test with Dunn’s multiple comparison test. Two groups were compared: Wilcox test. Each point represents one patient. *p < 0.05, **p < 0.01, ***p < 0.001 and not significant (ns). TLS, tertiary lymphoid structure; TC-TLS, TLS in the tumour core; TC-stroma, stromal regions in the tumour core; IM-TLS, TLS in the invasion margin; IM-Non-TLS, invasion margin excluding TLS; N-TLS, TLS in normal tissue; N-Non-TLS, normal tissue excluding TLS. TLS, tertiary lymphoid structure. Fig. S3. Survival analysis based on the effective scores. (A) PFS and OS of patients with GC based on the effective scores of tumour-infiltrating immune cells (TIICs). The effective scores were divided into high (>half of the patients; red line) or low density (≤half of patients; blue l [file 12916_2025_3889_MOESM1_ESM.docx]

**Supplementary Figures**

**Title:** Immune Microenvironment Spatial Landscapes of Tertiary Lymphoid Structures in Gastric Cancer

Supplementary Figures and Legends

**
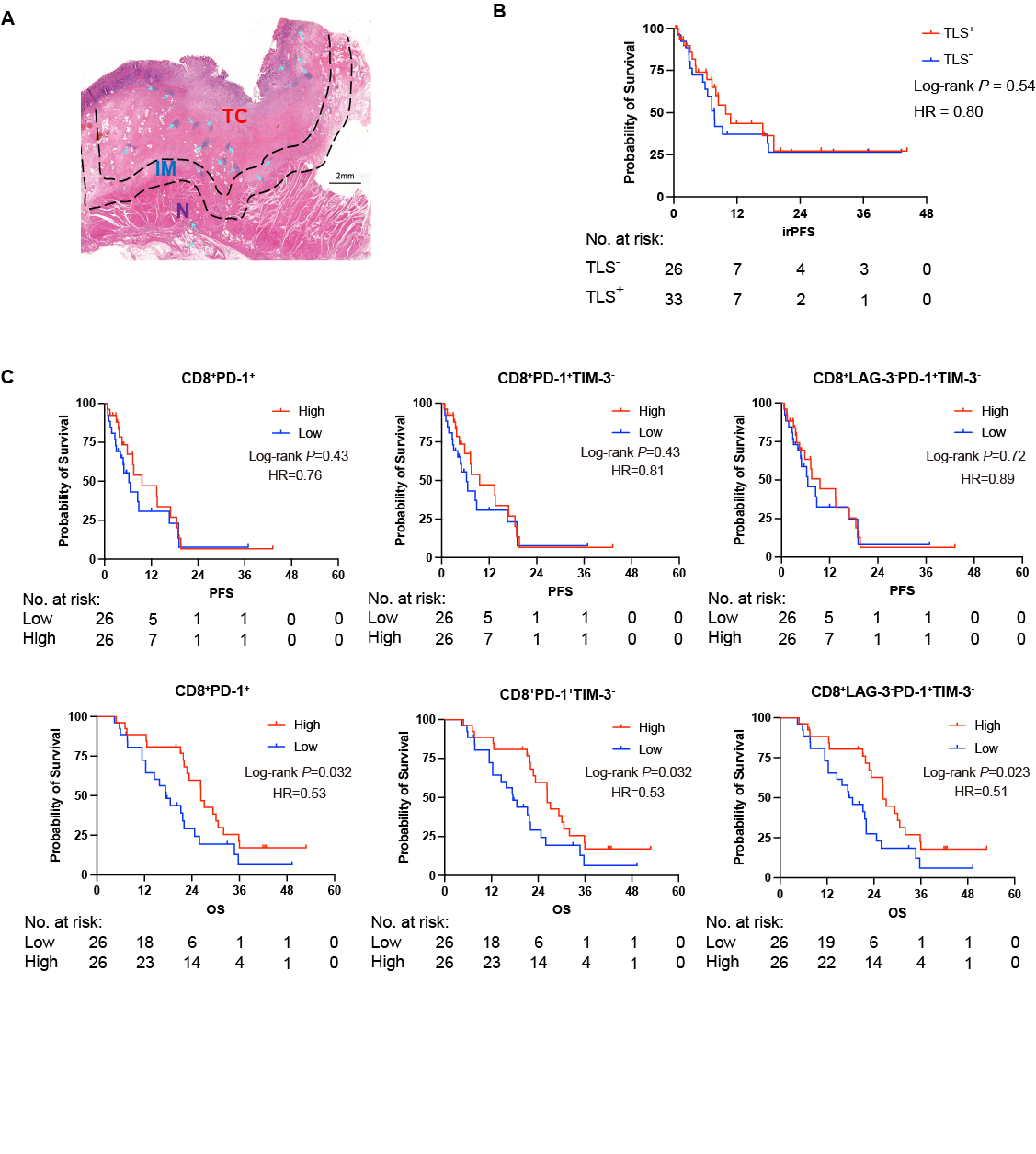
**

**Supplementary Fig. 1.**

(A) Selection of the regions of interest (ROIs) in representative images of haematoxylin and eosin (H&E)-stained formalin-fixed paraffin-embedded tissues. TC, tumour core; IM, invasion margin; N, normal tissue. Scale bar: 2 mm.

(B) Kaplan–Meier survival curves for irPFS of patients with and without TLS in the tumour core.

*P*-values are two sided.

(C) PFS and OS of patients with GC based on the density of tumour-infiltrating immune cells (TIICs). The individual TIICs were divided into high (>half of the patients; red line) or low density (≤half of patients; blue line). The log-rank (Mantel–Cox) test was used. A two-sided P < 0.05 was considered statistically significant.

GC, gastric cancer; TLS, tertiary lymphoid structure; irPFS, immune-related progression-free survival; PFS, progression-free survival; OS, overall survival.


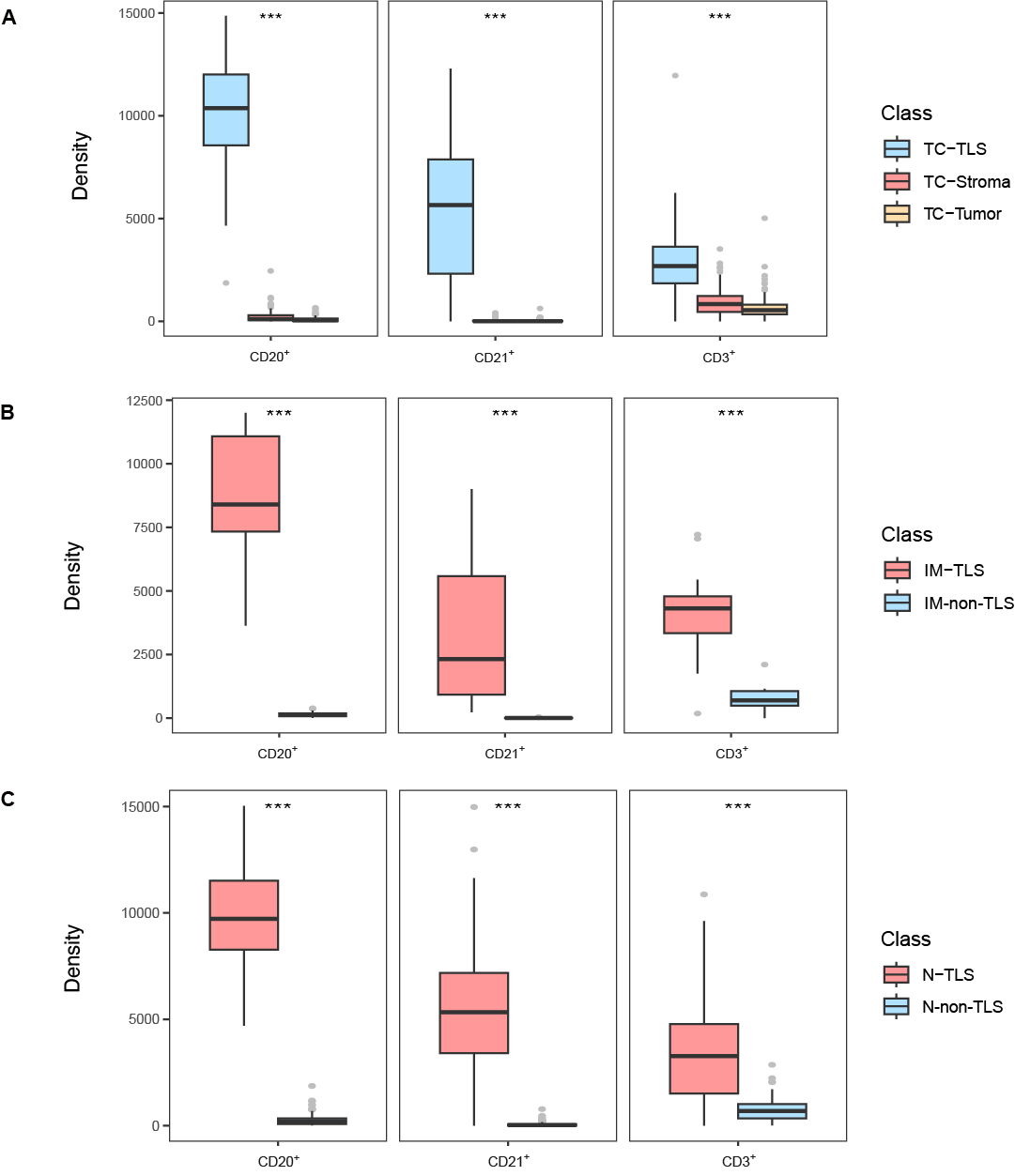


**Supplementary Fig. 2**.

(A–C). The density of main cell types of tumour core (A), invasion margin (B) and normal tissue (C). Box and whiskers represent mean ± 10–90 percentile. Three groups were compared: Kruskal–Wallis test with Dunn’s multiple comparison test. Two groups were compared: Wilcox test. Each point represents one patient. *p < 0.05, **p < 0.01, ***p < 0.001 and not significant (ns).

TLS, tertiary lymphoid structure; TC-TLS, TLS in the tumour core; TC-stroma, stromal regions in the tumour core; IM-TLS, TLS in the invasion margin; IM-Non-TLS, invasion margin excluding TLS; N-TLS, TLS in normal tissue; N-Non-TLS, normal tissue excluding TLS. TLS, tertiary lymphoid structure.


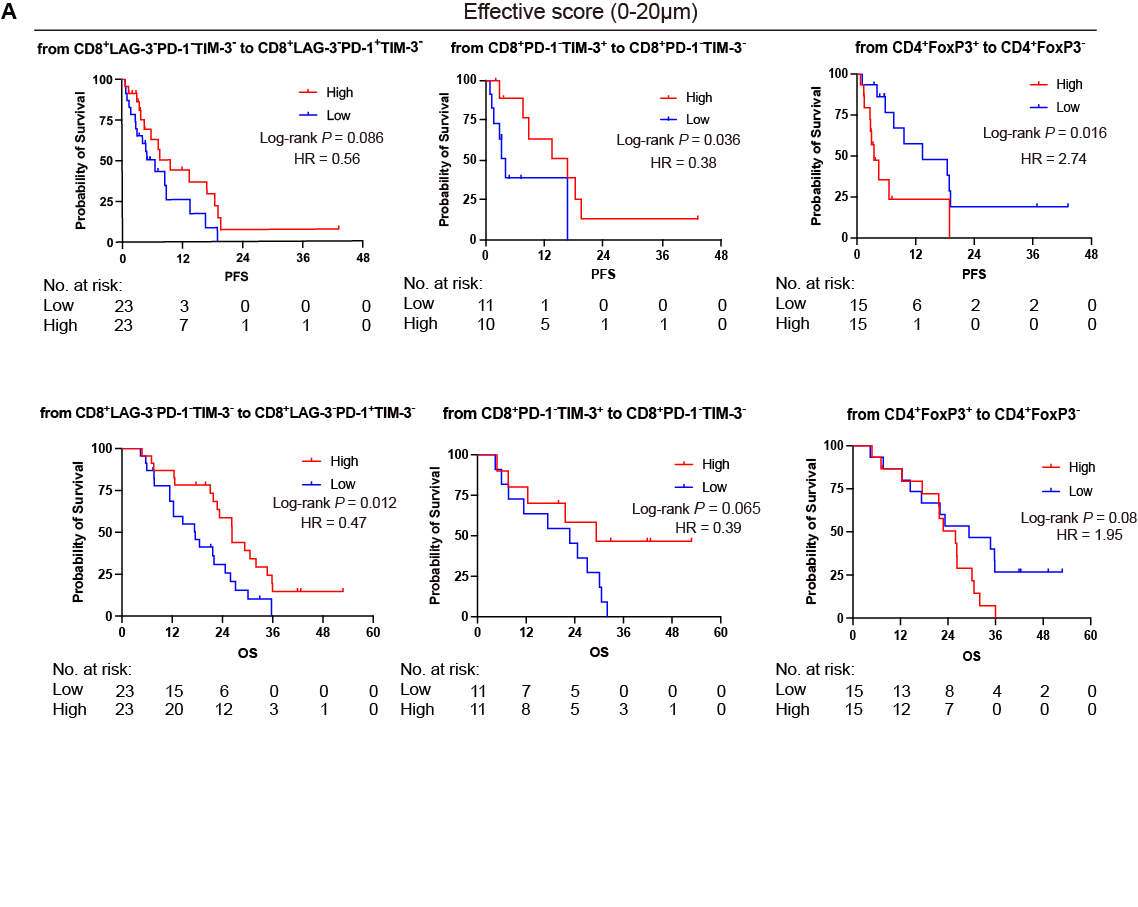


**Supplementary Fig. 3**.

(A) PFS and OS of patients with GC based on the effective scores of tumour-infiltrating immune cells (TIICs). The effective scores were divided into high (>half of the patients; red line) or low density (≤half of patients; blue line). Log-rank (Mantel–Cox) test was used. A two-sided p < 0.05 was considered statistically significant.

PFS, progression-free survival; OS, overall survival.

**
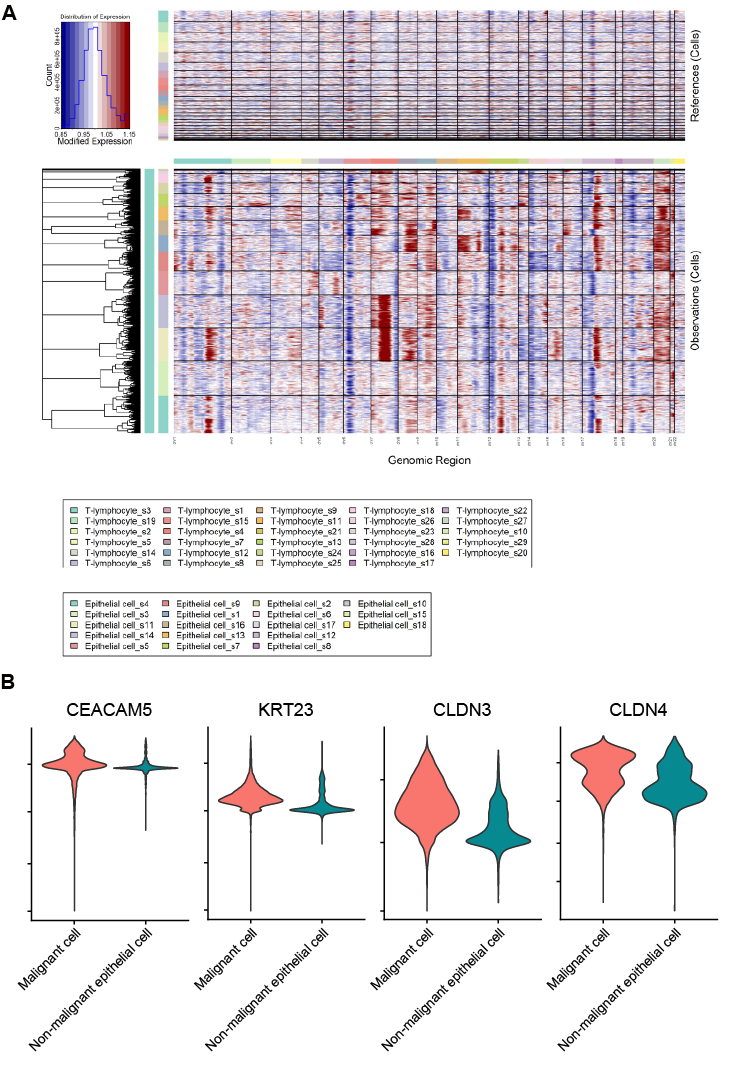
**

**Supplementary Fig. 4.**

1. The hierarchical heatmap showing large-scale CNVs in tumour lesions.
2. Violin plots for marker genes of cancer cells (*CEACAM5, KRT23, CLDN3*, and *CLDN4*).


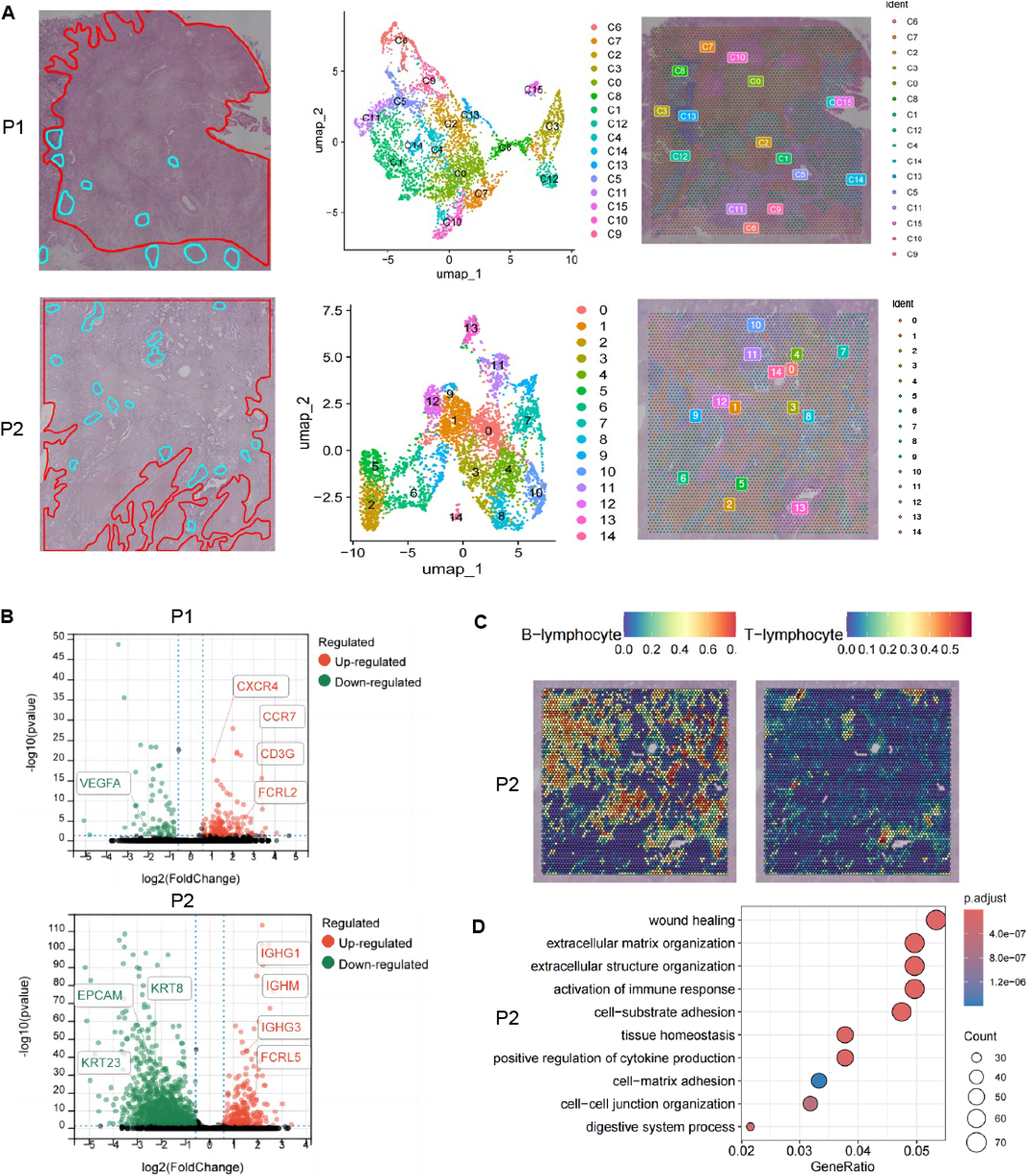


**Supplementary Fig. 5.**

(A) Left to right: Haematoxylin and eosin (H&E) stained tissues which are delineated into tumour (red) and TLS (blue), UMAP of the cluster identities, spatial distribution of the clusters in two cases of GC undergoing spatial transcriptomic sequencing.

(B) Volcano plot displayed the differentially expressed genes (DEGs) in TLS than the tumour regions.

(C) Spatial feature plots of signature score of B cells, and T cells in P2.

(D) Gene ontology (GO) analysis showing enriched biological process terms of DEGs in TLS than the tumour regions in P2. GC, gastric cancer; TLS, tertiary lymphoid structure; UMAP, Uniform Manifold Approximation and Projection.
