# Supplementary material for: Immune microenvironment spatial landscapes of tertiary lymphoid structures in gastric cancer
Source: BMC Med. 2025 Feb 4;23:59. doi: 10.1186/s12916-025-03889-3 (PMC11792408; doi:10.1186/s12916-025-03889-3)
Supplement: Supplementary file 2 — Additional file 2: Table S1. Summary of recurrence and distant metastasis in stage I–III patients (N=16). Table S2. The baseline characteristics of 110 gastric adenocarcinoma patients. Table S3. The baseline characteristics of gastric adenocarcinoma patients in spatial transcriptomic cohort (N=2). Table S4. The baseline characteristics of gastric adenocarcinoma patients in single-cell sequencing cohort (N=12). Table S5. Summary of primary antibodies and secondary antibody used for multiplex immunohistochemistry. [file 12916_2025_3889_MOESM2_ESM.docx]

Supplementary Tables

Table 1. Summary of recurrence and distant metastasis in stage I–III patients (N=16).

| Patient ID | Local recurrence | | Distant metastases | Sites of distant metastases |
| --- | --- | --- | --- | --- |
| Patient 1 |  | | Yes | Pleural, peritoneal, bone, ovary, distant lymph nodes |
| Patient 2 |  | | Yes | Liver, pancreas |
| Patient 3 | Yes | | Yes | Peritoneal |
| Patient 4 | Yes | | Yes | Distant lymph nodes |
| Patient 5 | Yes | | Yes | Peritoneal |
| Patient 6 | |  | Yes | Distant lymph nodes |
| Patient 7 | |  | Yes | Liver |
| Patient 8 | | Yes | Yes | Peritoneal, liver, pelvic cavity, distant lymph nodes |
| Patient 9 | |  | Yes | Pancreas |
| Patient 10 | | Yes | Yes | Peritoneal |
| Patient 11 | |  | Yes | Liver, distant lymph nodes |
| Patient 12 | |  | Yes | Distant lymph nodes |
| Patient 13 | |  | Yes | Liver |
| Patient 14 | |  | Yes | Liver |
| Patient 15 | |  | Yes | Liver, distant lymph nodes |
| Patient 16 | |  | Yes | Distant lymph nodes |

Supplementary Table 2. The baseline characteristics of 110 gastric adenocarcinoma patients.

| Characteristic | Total  (N=110) |
| --- | --- |
| Age at diagnosis |  |
| <65 | 59 (53.6) |
| ≥65 | 51 (46.4) |
| Gender |  |
| Male | 68 (61.8) |
| Female | 42 (38.2) |
| Stage at diagnosis |  |
| I | 2 (1.8) |
| II | 4 (3.6) |
| III | 29 (26.4) |
| IV | 75 (68.2) |
| Tumor differentiation |  |
| High | 15 (13.6) |
| Moderate | 39 (35.5) |
| Poor | 52 (47.3) |
| Unknown | 4 (3.6) |
| Lauren classification |  |
| Intestinal type | 50 (40.7) |
| Mixed type | 23 (25.4) |
| Diffused type | 34 (30.5) |
| Unknown | 3 (3.4) |
| Location |  |
| GEJ | 63 (57.3) |
| Non-GEJ | 47 (42.7) |
| HER2 expression |  |
| Positive | 20 (18.2) |
| Negative | 90 (81.8) |
| EBER expression |  |
| Positive | 6 (5.5) |
| Negative | 101 (91.8) |
| Unknown | 3 (2.7) |
| Microsatellite status |  |
| Microsatellite instability (MSI) | 12 (10.9) |
| Microsatellite-stable (MSS) | 98 (89.1) |
| PD-L1 CPS |  |
| CPS<1 | 41 (37.3) |
| 1≤CPS<5 | 15 (13.6) |
| 5≤CPS<10 | 12 (10.9) |
| CPS>10 | 38 (34.5) |
| Unknown | 4 (3.6) |
| Line of therapy |  |
| 1 | 61 (55.5) |
| ≥2 | 49 (44.5) |

Abbreviations: GEJ, gastro-esophageal junction; EGFR, epidermal growth factor receptor; HER2, human epidermal growth factor receptor 2; CPS, combined positive score; PD-1, programmed cell death protein 1; PD-L1, programmed death-ligand 1.

Supplementary Table 3. The baseline characteristics of gastric adenocarcinoma patients in spatial transcriptomic cohort(N=2).

| Patient ID | Sex | Age | Location of the primary tumor | Lauren Classification | Differential |
| --- | --- | --- | --- | --- | --- |
| Patient 1 | Male | 42 | Non-GEJ | Diffuse | Low |
| Patient 2 | Male | 54 | Non-GEJ | Intestinal | Moderate |

Abbreviations: GEJ, gastro-esophageal junction; TLS, tertiary lymphoid structure.

Supplementary Table 4. The baseline characteristics of gastric adenocarcinoma patients in single-cell sequencing cohort(N=12).

| Patient ID | Sex | Age | Location of the primary tumor | Lauren Classification | Differential | Presence of TLS |
| --- | --- | --- | --- | --- | --- | --- |
| Patient 1 | Male | 64 | Non-GEJ | Mixed | Low | Yes |
| Patient 2 | Male | 68 | Non-GEJ | Diffuse | Low | No |
| Patient 3 | Male | 76 | GEJ | Diffuse | / | Yes |
| Patient 4 | Female | 71 | GEJ | Intestinal | Low | No |
| Patient 5 | Male | 65 | GEJ | Intestinal | Moderate | No |
| Patient 6 | Male | 76 | GEJ | Intestinal | Moderate | No |
| Patient 7 | Male | 73 | GEJ | Intestinal | Moderate | No |
| Patient 8 | Male | 60 | GEJ | Intestinal | Low | No |
| Patient 9 | Male | 76 | GEJ | Intestinal | Low | No |
| Patient 10 | Male | 78 | Non-GEJ | Intestinal | Low | Yes |
| Patient 11 | Male | 61 | Non-GEJ | Mixed | Low | No |
| Patient 12 | Female | 72 | Non-GEJ | Mixed | Low | Yes |

Abbreviations: GEJ, gastro-esophageal junction; TLS, tertiary lymphoid structure.

| Supplementary Table 5. Summary of primary antibodies and secondary antibody used for multiplex immunohistochemistry. | | | |
| --- | --- | --- | --- |
| Antibodies | Source | Identifier | Dilution |
| CD8 | Cell Signaling Technology | CST70306 | 1:400 |
| LAG-3 | Cell Signaling Technology | CST15372 | 1:200 |
| TIM-3 | Cell Signaling Technology | CST45208 | 1:300 |
| PD-1 | Cell Signaling Technology | CST86163 | 1:200 |
| PanCK | Cell Signaling Technology | CST4545 | 1:500 |
| CTLA-4 | Abcam | ab237712 | 1:200 |
| CD4 | Biolynx | BX22300130 | 1:2000 |
| FoxP3 | Biolegend | BLG320202 | 1:80 |
| PD-L1 | Cell Signaling Technology | CST13684 | 1:800 |
| STING | Cell Signaling Technology | CST13647 | 1:2000 |
| CD68 | ZSGB-BIO | ZM0060 | 1:500 |
| CD163 | Cell Signaling Technology | CST93498 | 1:500 |
| CD86 | Cell Signaling Technology | CST91882 | 1:200 |
| CD66b | Gene Tex | GTX19779 | 1:400 |
| CD21 | Abcam | ab75985 | 1:1000 |
| CD56 | Cell Signaling Technology | CST3576 | 1:200 |
| CD20 | Abcam | ab78237 | 1:1000 |
| CD3 | Abcam | ab135372 | 1:400 |
| α-SMA | Abcam | ab5694 | 1:400 |
| FAP-α | Abcam | ab207178 | 1:500 |
| Horseradish-peroxidase-conjugated anti-rabbit and anti-mouse antibody | PANOVUE | 10013001050 |  |
